# Revisiting ABC Transporters and Their Clinical Significance in Glioblastoma

**DOI:** 10.3390/ph18010102

**Published:** 2025-01-15

**Authors:** Brandon Wee Siang Phon, Shalini Sundramurthi Chelliah, Dina El-Rabie Osman, Saatheeyavaane Bhuvanendran, Ammu Kutty Radhakrishnan, Muhamad Noor Alfarizal Kamarudin

**Affiliations:** 1 Jeffrey Cheah School of Medicine and Health Sciences, Monash University Malaysia, Jalan Lagoon Selatan, Bandar Sunway 47500, Selangor, Malaysia; brandon.phon@monash.edu (B.W.S.P.); shalini.sundramurthichelliah@monash.edu (S.S.C.); dina.osman@monash.edu (D.E.-R.O.); bsaatheeyavaane.bhuvanendranpillai@monash.edu (S.B.); ammu.radhakrishnan@monash.edu (A.K.R.); 2 Department of Biochemistry and Molecular Biology, Monash University, Melbourne, VIC 3800, Australia

**Keywords:** glioblastoma, ABC transporters, drug transport, increased drug efficacy, temozolomide, alkylating agents

## Abstract

**Background**: The multiple drug-resistant phenomenon has long since plagued the effectiveness of various chemotherapies used in the treatment of patients with glioblastoma (GBM), which is still incurable to this day. ATP-binding cassette (ABC) transporters function as drug transporters and have been touted to be the main culprits in developing resistance to xenobiotic drugs in GBM. **Methods**: This review systematically analyzed the efficacy of ABC transporters against various anticancer drugs from 16 studies identified from five databases (PubMed, Medline, Embase, Scopus, and ScienceDirect). **Results**: Inhibition of ABC transporters, especially ABCB1, improved drug efficacies. Staple GBM phenotypes, such as GBM stem cells and increased activation of the PI3K/Akt/NF-κB pathway, have been implicated in the expression of several ABC transporters. Using the datasets in The Cancer Genome Atlas and Gene Expression Omnibus, we found upregulated ABC transporters that either negatively impacted survival in univariate analyses (ABCA1, ABCA13, ABCB9, ABCD4) or were independent negative prognosis factors for patients with GBM (ABCA13, ABCB9). Our multivariate analysis further demonstrated three ABC transporters, ABCA13 (Hazard Ratio (HR) = 1.31, *p* = 0.017), ABCB9 (HR = 1.26, *p* = 0.03), and ABCB5 (HR = 0.77, *p* = 0.016), with the administration of alkylating agents (HR = 0.41, *p* < 0.001), were independent negative prognosis factors for patients with GBM. Conclusions: These findings reinforce the important role played by ABC transporters, particularly by ABCA13, ABCB9, and ABCB1, which could be potential targets that warrant further evaluations for alternate strategies to augment the effects of existing alkylating agents and xenobiotic drugs.

## 1. Introduction

**Figure 1:**
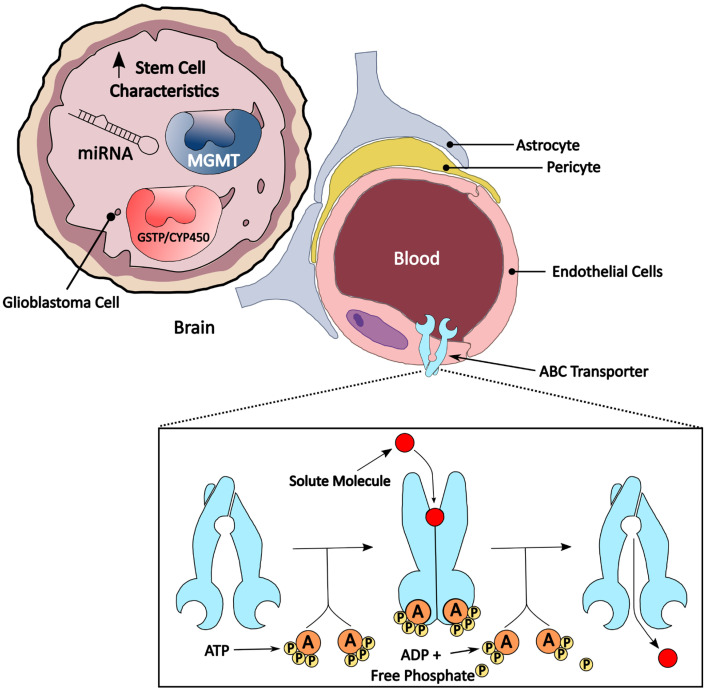
The depiction of the MDR phenomenon in GBM tumors. Drug resistance in GBM can be attributed to multiple factors, such as the presence of the BBB, ABC transporters that are highly expressed on the membranes of endothelial cells of the BBB, the presence of O6-methylguanine-DNA methyltransferase (MGMT), the presence of drug-metabolizing enzymes, increased stem cell characteristics in GBM cells, and the presence of miRNAs, to name a few. The presence of the BBB forms the first barrier of entry for most drugs due to restricted paracellular diffusion. Alkylating agents that cross the BBB into the cells will have to contend with the highly expressed suicidal MGMT enzyme in GBM that is capable of reversing DNA damage inflicted upon the tumor. Furthermore, GBMs often rely on miRNAs to regulate the transcription of cell death-related genes, tumor suppressor genes, and even the transcription of ABC transporters. Drugs that do penetrate the cells are often metabolized by enzymes such as glutathione S-transferases (GSTs) or cytochrome P450s (CYP450), expediting the excretion process. Finally, drugs that cross the BBB will have to contend with the efflux actions of ABC transporters.

Notorious for its low overall survival rate, glioblastoma (GBM) is one of the most common brain neoplasms among adults. A combination of aberrant mitotic activities, extensive necrotic regions, microvascular proliferation, and nuclear atypia defines GBM’s histology, with high motility, increased resistance to therapy, and high tumor heterogeneity defining their aggressiveness [1]. Furthermore, the standard therapy against GBM, known as the Stupp protocol, which includes concomitant treatment with radiotherapy and chemotherapy with temozolomide (TMZ), has provided only meager improvement to patients’ quality of life, extending the median survival of patients by an additional two months from the initial 12.1 months [2]. Widespread efforts to incorporate targeted therapies or immunotherapies as adjuvant therapies to the standard therapy for GBM were met with varying results. The lack of advancement in GBM treatments has been attributed to the inherent genetic and cellular heterogeneity of GBMs, which are characterized by the presence of stem-like cells and the presence of various neoplastic stromal cells [3]. The inherent heterogeneity of GBM has been correlated to the Multiple Drug Resistance (MDR) phenomenon, which may either be intrinsically present from the start of the therapy or acquired throughout the course of the therapy [1]. The mechanisms behind MDR are multifactorial (Figure 1), which include genetic regulation of mechanisms/pathways that inhibit apoptosis but promote proliferation; increased drug efflux; increased drug detoxification and metabolism; and increased DNA repair systems [1].

ABC transporters belong to one of the most conserved superfamilies, which consists of seven subfamilies (ABC-A to -G) spanning 49 proteins [4]. ABC transporters play multiple biological roles, ranging from cholesterol homeostasis, lipid trafficking, and myelin formation to forming part of the peptide-loading complex to deliver cytosolic peptides to nascent MHC class I molecules, being involved in mRNA translation, and functioning as peroxisome transporters [5]. ABC transporters export ligands from ions, peptides, lipids, and small proteins across cellular membranes by utilizing energy from the hydrolysis of adenosine triphosphate (ATP) [6]. The mechanism of action of ABC transporters involves a nucleotide-binding domain that relies on the hydrolysis of ATP to drive conformational changes in the transmembrane domain to allow solute molecules to cross the lipid bilayer of the membrane (Figure 1) [7]. It has been proposed that the high expression of ABC transporters in various cancer tissues [8], including GBM, which contribute greatly to the efflux of numerous xenobiotics across the cellular membrane, has made them a key target in MDR regulation [5,9,10,11]. Several studies suggested that ABC transporters may be related to negative clinical prognosis in ovarian [5], breast [12,13], colon [14,15], liver [16,17,18], skin [19,20], and brain [21,22] cancers.

As an exporter, ABC transporters can influence the inflammatory response by transporting paracrine hormones such as prostaglandins and leukotrienes or even interleukin-1β from cancer stem cells, culminating in activations of extracellular G-protein-coupled receptors or cytokine receptors, creating microenvironments that promote survival, angiogenesis, and migration [23]. Similarly, these exporters are capable of transporting glutathione, which is essential for cellular redox homeostasis and the removal of free radicals produced by radiation therapies in the presence of oxidative stress [23].

Brain uptake of therapeutic drugs has always been complicated and limited by the presence of the blood–brain barrier (BBB) that forms a well-defined intracranial environment. The BBB is mainly made up of endothelial cells, pericytes, astrocytes, microglia, and the extracellular matrix, which forms a physical and biological barrier [24,25] (Figure 1). Pericytes, astrocytes, and the microglia that form the neurovascular unit form a dynamic system with the neurons and the vascular system to transduce biochemical and biomechanical signals. This dynamic interaction forms one of the backbones behind the physical, biochemical immune barriers of the central nervous system [25]. The presence of tight junctions and adherens junctions in the endothelial cells severely hampers the passive paracellular diffusion of xenobiotic molecules. The endothelial cells present in the BBB practically restrict passive paracellular diffusion of drugs to only those that are soluble in lipids, and this passive diffusion has to also bow down to the whims of the blood/brain concentration gradient. Furthermore, endothelial cells of cerebral vessels carry the responsibility of providing protection in the BBB against drugs in the blood circulation via ABC transporters [24]. These specialized endothelial cells highly express ABCB1 (AKA P-glycoprotein) and ABCG2 (breast cancer resistance protein (BRCP), which are responsible for the efflux of chemotherapeutic drugs out of the brain parenchyma. The side population phenotype that is characterized by increased expressions of ABC transporters and drug efflux capacities is perfectly exemplified by the endothelial cells in the BBB [26].

ABC transporters and their affinity for a wide range of chemotherapeutic drugs remain a significant stumbling block toward increased quality of life in patients with GBM. Active programs were launched in the eighties and nineties that aimed to reverse the MDR phenotype of GBMs via the concomitant use of chemotherapeutic agents and specific ABCB1 inhibitors [27]. In theory, this was a sound strategy to increase drug accumulation in the brain parenchyma without compromising the BBB. However, efforts on that front have since died down following failed clinical trials at reversing the MDR phenotype in solid tumors due to high toxicities [28]. Some of the reasons cited for the decreased attention on ABC transporters were related to the multifactorial mechanisms of MDR that cannot be solved by a single modality [29].

More recently, the legitimacy of ABC transporter inhibitions has resurfaced with the advent of incremental empirical evidence in preclinical settings. Furthermore, in three-dimensional GBM cell cultures that more accurately mimic in vivo GBM settings, ABC transporters were markedly upregulated [30], an indication that ABC transporters are an acquired resistance that requires closer attention. The finding that ABC transporters could be predictors of a lesser favorable outcome has only fanned the flames of this debate [30]. Establishing the relationship between the presence of ABC transporters and drug efficacy in GBM using more advanced genetics and precision medicine approaches can affirm their role in the therapy of patients with GBM. Thus, this review aims to review and analyze the available evidence to investigate if inhibition of ABC transporters can be a viable strategy to improve the quality of life of patients with GBM. Additionally, through this review, we aim to provide answers to alternative strategies for ABC transporter inhibitions as well as identify specific ABC transporters for future scrutiny.

## 2. Results

### 2.1. Literature Search Results

**Figure 2:**
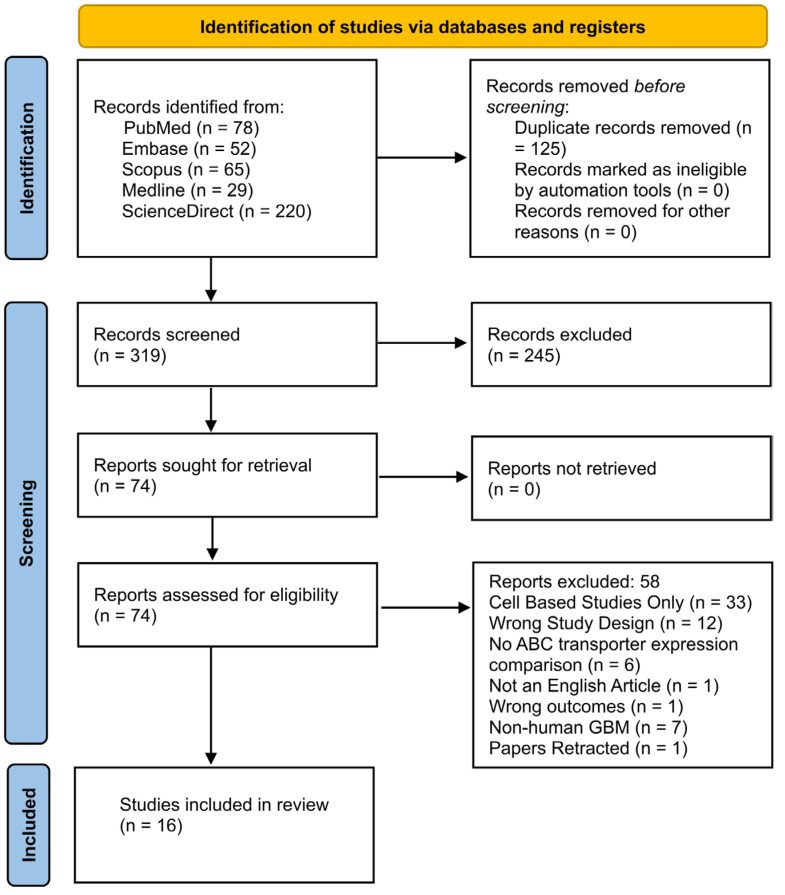
PRISMA flow chart depicting the entire literature search and study selection process.

The search from the five electronic databases yielded a total of 313 references, which were exported to Covidence for the screening process after the removal of 123 duplicates (Figure 2). The title and abstract screening yielded 74 articles that were deemed to be eligible for the full-text review for another round of eligibility assessment based on the inclusion and exclusion criteria of this study. During the full-text review, 58 articles were excluded based on our exclusion criteria, leaving 16 studies, which were included in the review.

### 2.2. Experimental Setups of Included Studies

The experimental setups for all the studies are delineated in Table 1. Various types of GBM cell lines were used for the in vitro studies (Table 1). The most prominent human GBM cell lines used in these studies were U-87MG (10 studies) [31,32,33,34,35,36,37,38,39,40], followed by patient-derived cultures (PDC) (8 studies) [31,34,35,37,41,42,43,44]. A-172 cells [32,33,37,44] were used in 4 studies, while U-251MG [34,35,40] and LN-229 [36,38,44] cell lines were used in 3 different studies. Lastly, LN-18 [38,44] and T98G [32,39] cell lines were used in 2 studies, while A1207 [44], LN-443 [37], U-118MG [37], and GBM8401 [40] cell lines were used in one study, respectively. A similar utilization of GBM cell lines was observed in the in vivo studies, where 10 studies used U-87MG cells [31,32,33,34,36,37,38,39,40,45], 4 studies used patient-derived cultures [41,42,43,46], while U-251MG [35], A1207 [44], GBM8401 [40], LN-229 [38], and T98G cells [39] were all used in one study, respectively.

ABCB1 was the most studied transporter across the 17 studies, being included in 8 in vitro studies and 9 in vivo studies [32,34,37,39,41,42,43,45,46]. ABCG2 [35,41,44] and ABCC1 [31,36] were reported in three and two in vitro and in vivo studies, respectively. ABCC3 [44], ABCC6 [44], ABCB5 [38], and ABCE1 [33] were all reported in one in vitro and in vivo study [31], respectively, while ABCA2 [44] and ABCC2 [44] was reported in one in vitro study.

In terms of drugs that were used, temozolomide (TMZ) (10 in vitro studies, 11 in vivo studies) stands out as the most studied candidate [32,33,34,35,36,38,39,41,42,43,46]. The other drugs, such as doxorubicin, were studied in three in vitro studies [40,42,43] while carmustine was reported in two in vitro and in vivo studies [40,44]. Irinotecan was used in two in vitro studies [37,42] and one in vivo study [37]. Etoposide and topotecan were used in one in vitro study [42], while abemaciclib [45], palbociclib [45], and rucaparib [46] were used in one in vivo study, respectively. Vincristine [31], aldoxorubicin [32], pitavastatin [37], and erlotinib [39], on the other hand, were used in one in vitro and one in vivo study.

### 2.3. Inhibition of ABC Transporters Increases Drug Efficacy In Vitro

Table 2 illustrates the increased efficacy of different drugs when ABC transporters are either inhibited or downregulated in the presence of ABC inhibitors, with TMZ being the most prominent drug [33,34,35,36,38,39,41,42,43]. Zhang et al. and Lee et al. reported that inhibition of ABCE1 and ABCB5 increased TMZ-induced apoptosis [33,38]. Furthermore, a further decrease in proliferation [41] and cell viability [34,36,39,42,43] was reported in the presence of TMZ upon the inhibition of ABCB1, ABCG2, and ABCC1. TMZ IC_50_ was also decreased upon ABC transporter inhibition by multiple groups with different inhibitors. One group found inhibition of *ABCE1* via shRNAs decreased TMZ IC_50_ in U-87MG and A172 cells by half [33]. Zhang et al. reported similar findings, where ABCG2 downregulation in the presence of fasudil decreased TMZ IC_50_ in U-87MG, U-251MG, and patient-derived cultures by at least half [35]. Increasing the concentration of fasudil further decreased TMZ IC_50_, where they reported a ≥90% decrease in the half-maximal inhibitory with 10 µM of fasudil [35]. Decreased ABCB1 activity also led to increased TMZ, doxorubicin, etoposide, topotecan, and irinotecan potencies [37,42,43]. Meanwhile, Torres et al. reported increased drug efficacy for vincristine upon ABCC1 expression inhibition [31]. In another study, it was shown that inhibition of ABCC3 and ABCC6 correlated to increased carmustine efficacy [44]. Jiang et al. also reported the reduction of IC_50_ in irinotecan, where the concurrent introduction of pitavastatin inhibited ABCB1 glycosylation, attenuating the function of the efflux pump despite an increase in ABCB1 mRNA copies [37].

ABC transporter activity could also be manipulated without the presence of direct or genetic interference. For instance, Ros et al. found that ATP consumption by ABCB1 was equal to or less than the basal control in the presence of TMZ and aldoxorubicin, citing negative modulations of ABCB1 by TMZ as a possible mechanism that led to a significant decrease in cell viability compared to separate administrations of both drugs [32]. The treatment of GBM cells with the protein BMP7 also decreased the expression of *ABCB1* and *ABCG2* [41].

Tumor microenvironments can also affect ABC transporter expression. For instance, cycling hypoxia, a phenomenon where spatial and temporal fluctuations in oxygen levels occur, significantly increased ABCB1 protein and mRNA levels compared to those under uninterrupted hypoxia stress. This was reflected in Chou et al.’s findings, where it was revealed that HIF-1α was a critical transcriptional activator for *ABCB1* [40]. By simulating the condition through cycles consisting of 0.5–1% O_2_ for one hour interrupted by 5% CO_2_ and air for 30 min, Chou et al. managed to show decreased doxorubicin and carmustine efficacies, where cells treated with both drugs under cycling hypoxia consistently displayed higher cell viabilities than those under normoxia or non-interrupted hypoxia [40].

### 2.4. Increased Drug Efficacy Replicated In Vivo

Many of the key findings reported using cell-based studies were replicated in vivo studies (Table 2). Similar ABC transporter inhibition methods that were utilized in vitro continued the trend of increased drug efficacy in GBM xenografted mice or rats (Table 3 and Table 4). For TMZ specifically, the inhibition of ABCB1, ABCG2, ABCE1, ABCC1, and ABCB5 has seen significant decreases in either GBM tumor volume/weight by (~40–90% reduction) [33,34,35,36,38,39,41,42,43], or significant increases in median survival (~1.3- to 5-fold increase) [34,35,42,43]. Torres et al. disclosed similar findings where decreased ABCC1 expression correlated to a 75% decrease in GBM tumor size when treated with vincristine [31]. The potency of carmustine (~2-fold increase in median survival) [40] and irinotecan (79% decrease in tumor volume) [37] also increased in vivo when ABCB1 expressions or activities dwindled. Similarly, the concurrent administration of aldoxorubicin and TMZ not only reduced tumor sizes and improved median survival in GBM xenograft mice but also delayed mortality [32].

Identical mechanisms that inhibited ABC transporter expression in vitro similarly decreased ABC transporter expression in vivo (Table 4). The use of the carbonic anhydrase XII (CAXII) inhibitor, Ad-DKK3, fasudil, and MRS1220 reduced the expression of ABCB1, ABCG2, and ABCC1 in GBM-xenografted mice, respectively [31,35,42]. The use of shRNAs, micro-RNAs, and monoclonal antibodies that directly inhibited ABCE1, ABCC1, and ABCB5 in vivo was followed by decreased tumor growth [33,36,38]. Jeon’s research group noted the positive correlation between ID4 levels and ABCC3 and ABCC6 mRNA levels [44]. Increased ID4 levels resulted in increased SOX2 levels, resulting in decreased carmustine efficacy. They showed that mice that overexpressed ID4 and SOX2 had significantly higher tumor weight after carmustine treatment when compared to wild-type mice [44].

Furthermore, pre-treatment of EGFR inhibitor erlotinib in mice resulted in a significant reduction in ABCB1 expression [39]. By repressing HIF-1α directly using YC-1, Chou et al. managed to not only reduce ABCB1 expression in mice xenografted with U87MG and GBM8401 cells but also significantly increase its survival when administered concurrently with carmustine and doxorubicin [40].

### 2.5. Correlation of ABC Transporters and Increased Drug Accumulation In Vitro and In Vivo

The inhibition of ABC transporters revealed increased drug accumulation in GBM cells. Salaroglio et al. and Mujumdar et al. revealed an increase in intracellular TMZ accumulation when ABCB1 was inhibited using CAXII inhibitors [42,43]. Salaroglio et al. found that the introduction of CAXII inhibitors increased intracellular TMZ by approximately 1.5× while ABCB1 knockdown doubled the intracellular TMZ concentration [42]. Likewise, Mujumdar et al. found that decreasing ABCB1 activity using CAXII inhibitors increased the intracellular TMZ concentration in patient-derived cultures by at least 2-fold [43]. Rhodamine 123, a ligand of ABCB1, had decreased intracellular accumulation in cells under cycling hypoxia stress compared to cells under normoxia [40]. The introduction of HIF-1α siRNA that inhibited ABCB1 expression, on the other hand, significantly increased rhodamine 123 accumulation [40].

In vivo settings, inhibition of ABCB1 was found to increase the accumulation of drugs in the brain [45]. The absence of ABCB1 increased the brain-to-plasma ratio of both abemaciclib and palbociclib, which was observed in *ABCB1* knockdown mice [45]. This allowed enhanced BBB penetration of palbociclib, which was severely hampered by ABCB1 efflux [45]. Rucaparib, a ligand for both ABCB1 and ABCG2, also had significantly higher brain accumulation in mice that had total knockouts of both genes when compared to wild-type mice, increasing both the rucaparib concentration in the brain and the rucaparib brain-to-plasma ratio [46].

### 2.6. Association of ABC Transporter Expression with Chemotherapeutic Drugs and Major Implicated Pathways

Other than the increased genetic mutation and DNA repair mechanisms, the highly resistant nature of GBMs toward chemotherapeutic drugs could also be attributed to increased ABC transporter expressions. It was found that ABCB1, *ABCB5*, and ABCE1 expression was significantly higher in GBM compared to non-neoplastic brain tissues [33,34,38]. Additionally, it was revealed that the increased presence of *ABCB5* in three different GBM subtypes (proneural, mesenchymal, and classical) and decreased *ABCB1* transcription levels correlated to significantly lower overall survivals [37,38]. The issue is exacerbated by the fact that TMZ, the most common chemotherapeutic drug used against GBM, seems to have the ability to induce ABCB1 expression. Munoz et al. revealed that initial in vitro TMZ treatments led to increased expression of a transcription factor that can be activated by the JNK or ERK1/2 pathway, AP-1 [39]. This was prompted by the increased release of EGF and its receptor, which correlated to an increase in total and phosphorylated c-Jun and c-Fos [39]. Other drugs, such as irinotecan and pitavastatin, also increased intracellular *ABCB1* mRNA copies when administered [37]. However, Tso et al. reported contradictory findings, where they found a decrease in *ABCB1* expression in GBM stem cells treated with TMZ compared to untreated GBM stem cells [41].

**Figure 3:**
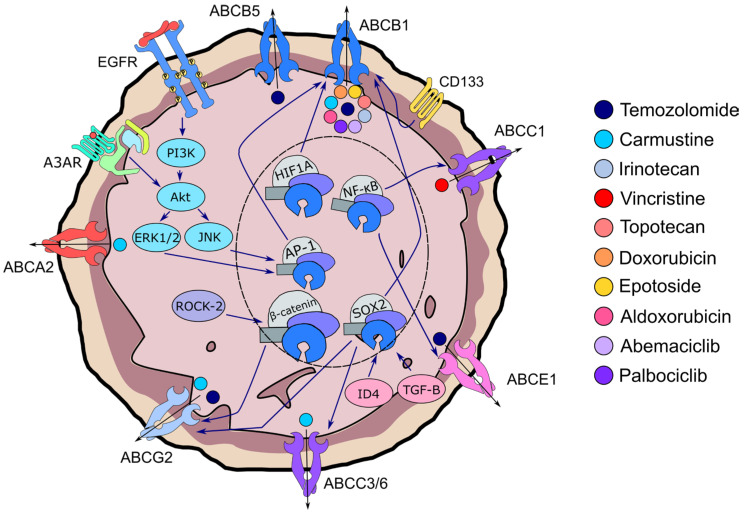
A summary of the drugs transported by the various ABC transporters reported and the signaling mechanisms that were hypothesized to be involved in ABC transporter regulations across the 17 included studies. Blue arrows indicate the potential signaling pathways involved, while the black arrows indicate the export of drugs by the ABC transporters.

A closer inspection of the pathways involved in ABC transporter expression revealed the possible redundancy in its activation mechanism (Figure 3). As mentioned above, *ABCB1* can be transcriptionally activated via the release of EGF and AP-1. Several findings have also corroborated the transcriptional activation of ABC transporters via the PI3K/Akt/NF-κB pathway [31,34]. Inhibiting the A3 adenosine receptor (A3AR) which belongs to the superfamily of G-protein-coupled receptors, resulted in a decrease in phosphorylated Akt and ERK1/2 expression, which ultimately led to a decrease in ABCC1 expression [31]. Similarly, Zhang et al. found that the blockade of the PI3K/Akt/NF-κB pathway enhanced the inhibitory effect on *ABCE1* expression. Furthermore, the administration of DKK3 proteins using adenovirus that decreased ABCB1 expression by Fujihara’s group was achieved via NF-κB inhibition [34].

Several other transcriptional mechanisms were also reported. For example, the use of fasudil inhibited Rho-associated kinase 2 (ROCK-2), which ultimately decreased ABCG2 expression [35]. This was made possible by the decreased phosphorylated moesin, decreased phosphorylated β-catenin, and decreased nuclear accumulation of β-catenin, upon ROCK-2 inhibition, where it was found that the regulation of ABCG2 expression was dictated by nuclear translocation of β-catenin [35]. SOX2, a notable GBM stem cell marker, was also revealed to be a transcription factor for *ABCC3* and *ABCC6*. It was found that the inhibition of ID4 lifts the repression of miR9, which is a repressor for the SOX2 gene [44]. The involvement of the aforementioned HIF-1α suggests that it too is a transcription factor for *ABCB1* [40]. Finally, Tso et al. revealed the downregulation of both *ABCB1* and *ABCG2* correlated to a decrease in *CD133* and *SOX2* expression when treated with bone morphogenetic proteins, likely via the suppression of TGF-β signaling [41].

### 2.7. Clinical Prognosis of ABC Transporters in the Cancer Genome Atlas (TCGA) GBM Cohort

**Figure 4:**
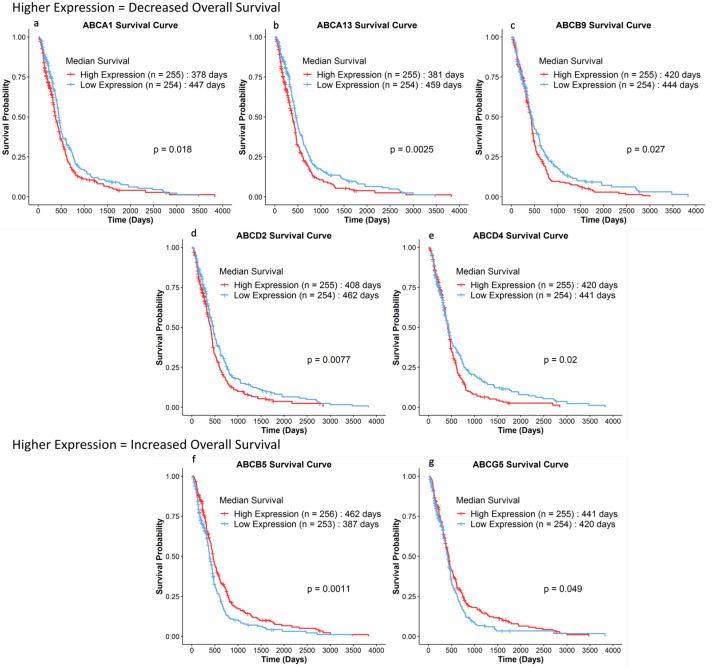
Kaplan–Meier Survival Curves of 509 patients with GBM that had an overall survival of more than 30 days based on the expression of ABC transporters. Patients were dichotomized into high and low-expression groups based on the median expression, with number of patients in each group stated in brackets. (**a**) *ABCA1*, (**b**) *ABCA13*, (**c**) *ABCB9*, (**d**) *ABCD2* and (**e**) *ABCD4* represent ABC transporters that have negative prognostic values within the 509 patients with GBM. (**f**) *ABCB5* and (**g**) *ABCG5* represent ABC transporters that have positive prognostic values within the 509 patients with GBM.

**Figure 5:**
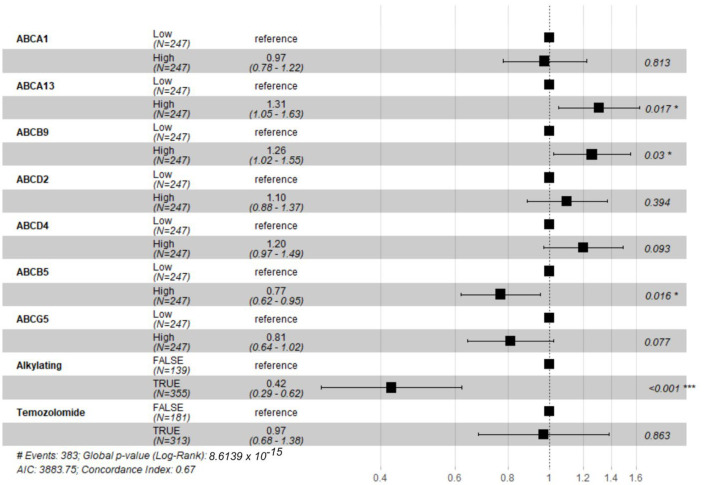
Forest plot of the multivariate survival analysis using Cox regression between ABC transporters that were statistically significant in the univariate analysis and the presence of alkylating agents or temozolomide in the treatment process. * represents a *p*-value that is less than 0.05; *** represents a *p*-value that is lower than 0.001.

It was established through the various literature that inhibiting ABC transporters in vitro and in vivo saw increased drug efficacies. In an attempt to resolve questions about their role in improving clinical outcomes and survival, we conducted Kaplan–Meier survival analysis of patients with GBM from TCGA to elucidate if this phenomenon extends to human patients with GBM as well. Overall survivals of 509 patients with GBM who survived more than 30 days were compared via log-rank tests based on the expression of 46 ABC transporters that were available in the TCGA dataset. Our results showed that higher expression of *ABCA1*, *ABCA13*, *ABCB9*, *ABCD2*, and *ABCD4* showed lower overall survival in patients (~21 to 78 days) (Figure 4). Interestingly, higher expression of *ABCB5* and *ABCG5* corresponded to an increase in overall survival (~21 to 75 days increase in the patients with the high-expression group compared to the low-expression group). To strengthen the correlation between the expression of ABC transporter, and drug efficacy, multivariate Cox regression analysis was carried out between the aforementioned ABC transporters, which were statistically significant, and whether the patients received alkylating agents or TMZ. Figure 5 represents the forest plot of the multivariate analysis, with *ABCA13* (Hazard Ratio (HR) = 1.31, *p* = 0.017), ACBC9 (HR = 1.26, *p* = 0.03), *ABCB5* (HR = 0.77, *p* = 0.016), and the usage of alkylating agents (HR = 0.42, *p* < 0.001) being independent prognostic factors.

In an attempt to further validate the results obtained from the survival analysis, we obtained data from seven different microarray datasets that performed gene expression comparisons between GBM samples and healthy brain samples and looked at the expression of the 7 ABC transporter, which statistically had an impact on patients with GBM survival. The number of samples in each dataset is denoted in Table 5. Of note, our differential expression analysis revealed that *ABCA1* was upregulated in patients with GBM across six different microarray datasets (Table 6). *ABCA13*, which was denoted as an independent prognostic factor in the multivariate survival analysis, was upregulated in patients with GBM across two different datasets. *ABCD4*, which was found to negatively affect survival in the univariate survival analysis, was upregulated in four different datasets. *ABCB9*, on the other hand, was only upregulated in one dataset, while three other microarray datasets denoted a decreased expression of the gene in patients with GBM.

## 3. Discussion

### 3.1. The Efficacy of Chemotherapeutic Drugs In Vitro and In Vivo in the Presence of ABC Transporters

One cannot deny that the advancement in the quality of life of patients with GBM has stagnated. However, this cannot be put down to the lack of effort, considering the bulk of work that has been dedicated towards the realization of new therapy paradigms. There are arguments to be made that instead of zeroing in on the classic anticancer properties such as initiating apoptosis, reducing proliferation, inducing cell cycle arrest, and restricting angiogenesis and migration via drug administration or genetic modulations, efforts should be diversified to mitigate GBM’s intrinsic resistance as well. The BBB has always been the first barrier to GBM treatments, forming a physical and biological barrier that reduces the brain’s exposure to drugs. Past the BBB, ABC transporters that reside in the BBB represent one of the other bottlenecks for efficient drug transport. A common consensus of the in vitro results is the increased drug efficacy when ABC transporters are either downregulated or inhibited. This statement rings true for various drugs, including widely used alkylating agents TMZ and carmustine, topoisomerase inhibitors etoposide, topotecan, and irinotecan; anthracyclines such as doxorubicin; as well as vincristine, procarbazine, and lomustine that are used in the PCV regimen [54,55,56,57].

Reports from the included studies corroborated the reliance of GBM tumors on ABC transporters when it comes to xenobiotic resistance. GBM cell lines that are more susceptible to alkylating agents such as A-172, U-87MG, U-251MG, LN-229, and A1207 [58,59] saw increased cell death upon carmustine and TMZ administration after ABC transporter inhibition. The underlying issue that has long since plagued TMZ and carmustine efficacy is the expression of MGMT. More importantly, TMZ and carmustine saw increased potency after ABC transporter inhibition in cell lines that are more resistant to alkylating agents, whether due to increased MGMT expression or due to the cells’ innate p53 mutation, such as LN-18, T98G, and GBM8401. Since MGMT participates in the DNA repair process in a suicidal reaction, an increase in TMZ exposure by virtue of decreased TMZ export by the GBM cells seems to be a potential strategy for increasing TMZ efficacy. Unfortunately, direct comparisons between chemosensitive GBM cells and chemoresistant cells were sparse in our included studies. Lee et al. reported increased ABCB5 expression in LN-18 compared to LN-229, with ABCB5 expression in U-87MG trumping both cell lines [38].

Initial exposure to drugs such as TMZ, pitavastatin, and irinotecan was found to induce the expression of ABCB1. This indicates that GBM tumors acquire drug resistance by initiating ABC transporter expression immediately as a means of survival against exposed xenobiotic molecules, which emphasizes the role of ABC transporters in conferring MDR. Munoz et al. noted a higher increase in acquired ABCB1 expression in T98G compared to U-87MG when GBM cells are exposed to TMZ [39]. It is apparent that ABC transporters are not the only factor in determining xenobiotic resistance in GBM cells, with how multifaceted MDR is. Regardless, ABC transporters still play a huge role in sensitizing GBM cells towards chemotherapeutic drugs, as evidenced by Zhang et al. when their group noted the sensitization of U-251MG and U-87MG that possessed generated TMZ resistance through multiple rounds of TMZ stimulation [33]. Findings from Salaroglio et al. [42] and Mujumdar et al. [43] corroborated the understanding that TMZ is indeed a ligand of ABCB1. While the lipophilic nature of TMZ allows it to diffuse across the BBB passively, being the ligand of a highly expressed ABC transporter counteracts its advantageous trait.

Six in vitro studies reported an increase in drug efficacy in neurospheres after ABC transporter modulation [31,35,41,42,43,44]. In vitro 2D cultures have come under some scrutiny for their inability to properly mimic actual tumor microenvironments properly owing to the lack of interaction between tumor cells, low resemblance of tumor phenotypes that is down to a different genetic landscape, and direct accessibility to oxygen, nutrients, and metabolites [60]. This might create scenarios where a drug’s effectiveness is overestimated in an adherent setting, which could have been a contributing factor to the low drug attrition rate in GBM therapy [61]. 3D cultures that are more reminiscent of GBM tumor phenotypes have been touted to provide a more accurate drug response in GBM cells [23]; hence the fact that ABC inhibition brings about increased drug efficacy brings added credibility to the reported in vitro results that would more accurately translate to a clinical setting. It was shown that intracellular TMZ accumulation was lower in neurospheres compared to adherent cultures [42,43]. Zhang et al. noted the increased TMZ IC_50_ in U-87MG neurospheres (3629.66 µM) compared to those cultured adherently (3262 µM), and inhibition of ABCG2 similarly decreased the TMZ IC_50_ in both culture methods (neurospheres: 236.43 µM, adherent: 176.16 µM) [35]. Finally, Torres’ research group reported increased ABCC1 expression in the GBM neurospheres compared to adherent culture, relating to the increased aggressiveness of GBM stem cells [31].

In vivo xenograft models also evade most of the adherent culture issues by replicating the setting of patients with GBM more accurately. More importantly, in vivo xenograft models can properly emulate the barrier to drug penetration that is the BBB, ensuring that any positive results reported would better translate to a clinical setting. The various observations recorded in vitro were also replicated in vivo, where the actual tumor microenvironment is more closely replicated. Inhibition of ABC transporters recorded increases in drug accumulations with increased TMZ, vincristine, carmustine, and irinotecan potency in xenograft mice (Table 3). While certain groups failed to report changes in ABC transporter activity/expression in vivo, similar results were reported when the same mode of inhibition used in vitro was applied in vivo. All these findings stipulate that even in an environment that closely resembles the human tumor microenvironment, drug penetration, and drug exposure can be enhanced by impeding the actions of ABC transporters [31,32,33,34,35,36,37,38,39,40,41,42,43,44]. The fact that GBM cells’ innate resistance towards drugs could be circumvented by virtue of increased drug exposure in vitro and especially in vivo is a strong attestation that the inhibition of ABC transporters is well worth investigating.

### 3.2. ABC Transporters and Their Associated Mechanisms

GBM exists as a tumor mass in the brain and is described as a three-layer concentric model, namely the peripheral layer, the intermediate layer, and the core [62]. This model is dictated by a hypoxia gradient, with the degree of hypoxia driving the state of dormancy of the cells going down the layers. GBM stem cells that inhabit the hypoxic core directly contribute to the increased chemotherapy resistance and the high relapse rate among patients with GBM [63]. The presence of GBM stem cells may promote GBM aggressiveness and resistance as observed by the high expression of SOX2, which acts as a transcription factor for *ABCC3* and *ABCC6* [43]. The fact that the expression of *ABCG2* and *ABCB1* was also found to be correlated with the expression of *SOX2* suggests that SOX2 acts as a transcription factor for multiple families of ABC transporters [41]. Additionally, *ABCB1* expression was significantly increased in the events of cycling hypoxia, stipulating that HIF-1α not only plays a part in maintaining the hypoxic niche within the GBM core but also acts as a transcription factor for *ABCB1* through a functional hypoxia-responsive element that exists in the *ABCB1* promoter [40]. The expression of *ABCG2* was also affected by the transcription factor β-catenin and the Wnt pathway, where decreased nuclear translocation of β-catenin was observed alongside the decrease in *ABCG2*. Β-catenin has been found to bind to the promoter of *ABCB1* [64]. The observation made by Zhang et al. [35] indicates that *ABCG2* may be one of the many downstream targets of β-catenin as well, an observation that was also reported in several other studies [65,66]. While the authors did not probe the effects of *CD133* and *SOX2* on *ABCB1* and *ABCG2* expression directly, the downregulation of both the ABC transporters accompanied the downregulation of both the stem cell markers through the introduction of BMP7. BMP7 is a member of the TGF-β superfamily of growth factors. The binding of BMP7 to its receptors suppresses the TGF-β signaling by blocking the nuclear translocation of phosphorylated SMADs 2/3 induced by TGF-β. This is achieved through the intervention of phosphorylation of intracellular SMAD1/5/8, induced by the binding of BMP7 to BMP type 1 and type 2 receptors [41]. Overexpression of the TGF-β signaling is well documented to induce stem cell properties through increased CD133 and SOX2 expression [67]. Studies have shown that CD133 can regulate the expression of ABCB1 through the PI3k/Akt/NF-κB pathway [68]. These findings further support the positive correlation between stem cell markers and the expression of ABC transporters [31,35,37,38,41,42,43,44].

Our included studies also revealed another strategy of targeting ABC transporters without relying on the ABC inhibitors, which brings about the issues of increased hematologic toxicities. Several studies reported on the involvement of the PI3K/Akt/NF-κB pathway in modulating the expression of ABCB1, ABCC1, and ABCE1 [31,33,39]. This redundancy in the expression pathways offers opportunities to solve the functionally overlapping nature of ABC transporters. Hematologic toxicities that come with ABC inhibitors can be potentially bypassed through genetic manipulations. As shown in Li et al.’s study, the use of miRNA interference is an effective strategy for inhibiting ABC transporters [36]. An avenue that is definitely worth considering, various miRNAs have been found to modulate the expression of ABCB1 (miR-145, −298, −331-5p, −451, −1253, −27a, −200c) and ABCG2 (miR-212, −328, −519c, −520h, −200c) [69]. Gene therapies such as the highly precise CRISPR-Cas9 gene editing tool can also be leveraged to specifically knock out the expression of ABC transporters [70]. Inhibition of several ABC transporters can be achieved through the inhibition of the PI3K/Akt/NF-κB pathway according to the observations in our included studies. Known to promote cell growth and survival, the PI3K/Akt/NF-κB pathway is one of the most highly upregulated pathways in GBM owing to the ubiquitous amplification of EGFRs and the infamous EGFRvIII mutation variant. EGFR inactivation and its downstream signaling pathways have been the research focus for most GBM researchers for a long time. The involvement of erlotinib in inhibiting the expression of ABCB1 further accentuates the variable roles played by this particular signaling pathway [39]. Any research on genetically manipulating ABC transporter expression could really benefit from first focusing on the PI3K/Akt/NF-κB pathway. A move that would not only be able to inhibit multiple ABC transporters at once, solving the functional overlapping issue of this resistance mechanism, but will also bring about multiple beneficial ramifications.

Ros et al. reported a peculiar way to tackle the issues of ABC transporters via the concurrent administration of temozolomide and aldoxorubicin [32]. Ros and co. hypothesized in their paper that the increased efficacy of both drugs was due to the negative modulation of ABCB1 by TMZ [32]. It was noted that the negative modulation was carried out via adverse effect on the ATPase activity of ABCB1, possibly down to competitive inhibition of saturated ABCB1s, without the need for any inhibitors or genetic interference. While effective in a preclinical setting, the effectiveness of such an approach is a concern since it would be highly dependent on nailing the dosage of the drugs administered without the risk of severe side effects. Considering the frequency of failed trials involving the concurrent administration of two or more anticancer drugs due to increased hematologic toxicities, this approach, while convenient, would require further scrutiny.

### 3.3. The Clinical Relevance of ABC Transporters in GBM

Seeking to determine the prognostic value of ABC transporters in patients with GBM, we obtained clinical data of patients with GBM from TCGA and performed some bioinformatics analysis. Our data showed that ABCA1, ABCA13, ABCB9, ABCD2, and ABCD4 carried a negative prognostic value when it comes to patients with GBM overall survival. Interestingly, none of the ABC transporters that were tested in our included studies, such as ABCB1 and ABCG2, seemed to carry any prognostic value in patients with GBM. However, the increased drug efficacy in vitro and especially in vivo cannot be ignored. The in vitro and in vivo studies coupled with the data from TCGA signify the overall preclinical and clinical importance of ABC transporters in patients with GBM. The multivariate analysis showed the increased hazard ratios of ABCA13 and ABCB9. Coupled with the decreased hazard ratio in patients with GBM receiving alkylating agents, the association seems to imply that the expression of these two transporters might lead to decreased efficacy for alkylating agents. The potential of ABCA13 is backed up by our differential expression analysis, where its gene was found to be upregulated in two different microarray datasets. Meanwhile, ABCB9 poses a more controversial figure. While ABCB9 was indeed upregulated in one study among patients with GBM [53], it was also found to be downregulated in patients with GBM across three other studies when compared to healthy brain samples [47,49,51]. This was reflected in the TCGA cohort as well with its low-expression values, suggesting that its impact might not be as significant as first seemed. While ABCA1 and ABCD4 failed to denote a statistically significant result in the subsequent multivariate analysis, the fact that both were upregulated across multiple microarray datasets (6 datasets for the former, 4 datasets for the latter) advocates for closer scrutiny in future GBM therapy evaluations. Not to mention, Yan et al. reported that *ABCA1* was not only associated with poorer overall survival in patients with glioma but also associated with enhanced TMZ resistance [71]. Besides the fact that the ABCA subfamily’s role as lipid transporters, the specific functions of ABCA1, ABCA13, and ABCD4 in cancer and specifically GBM are relatively unknown, which might warrant closer and more in-depth studies. Roy et al. recently published a report that looked at a cohort of 22 non-tumoral samples and 159 GBM tumor specimens and found that *ABCB1*, *ABCG2*, and *ABCC1* were significantly more expressed in GBM samples, with increased *ABCC1* correlating with a significantly shorter progression-free survival [72]. These findings, coupled with the translation of the in vitro results to an in vivo setting in the included studies, provide a solid framework for future studies that look to scale beyond the preclinical phase.

Interestingly, we noted two ABC transporters (ABCB5 and ABCG5) that were actually related to better overall survivals in the TCGA cohort, with ABCB5 even recording decreased hazard ratios in the multivariate Cox regression analysis. These results are somewhat surprising since ABCB5 and ABCG5 are related to chemoresistance in other cancers. The function of ABCB5 is unfortunately also unknown, but it is a biomarker that predicts the recurrence of oral squamous cell carcinoma [73] and is linked to chemoresistance in melanoma [74], liver cancer [75], breast cancer [76], and colon cancer [77]. The same can be said about ABCG5, which is a poor predictor of colorectal cancer [78] and is linked to increased xenobiotic resistance in head and neck cancers [79]. It has to be said that upon closer inspection of the microarray values, *ABCG5* in particular had largely negative log2 ratio values, indicating the downregulated status of *ABCG5* in most patients, with *ABCB5* similarly recording very low expression among the 509 patients. Therefore, it is plausible that *ABCG5* had no tangible impact on the survival of the patients. Further validation would definitely be required for the actual preclinical and clinical impact of ABCB5, and ABCG5.

### 3.4. Issues and Future Perspectives

A bird’s eye view of Table 4 will reveal the overlapping specificity of ABC transporters. This shines a light on the failures of the previous ABC transporter inhibition attempts. First approved clinically in 1981, previous attempts using ABC inhibitors either met with intolerable levels of toxicity or failed to make significant headway by not improving standard drug efficacy in multiple clinical trials [80,81,82,83,84,85,86]. For example, it seems TMZ is a ligand of five different ABC transporters among our included studies, while carmustine is a ligand of three different ABC transporters as well. Furthermore, our included studies indicated that ABCB1 is responsible for the efflux for every tested drug bar vincristine. The redundant nature of ABC transporters is reflected by the distinct binding sites on the transmembrane domain [7]. This creates a dilemma since this redundancy essentially renders a unified, one-size-fits-all approach practically impossible. This was one of the reasons behind the failed clinical trials of tariquidar (ABCB1 inhibitor) and elacridar (ABCB1 and ABCG2 inhibitor). Any improvements to the quality of life of patients with GBM using ABC inhibitors would have to seriously tackle the functionally overlapping nature of ABC transporters.

There is no doubt that ABC transporters are fascinating and attractive targets for improved GBM therapy. However, it would be disingenuous to ignore the function of ABC transporters in other parts of the human body. ABC transporters play a crucial role in the systemic clearance of xenobiotic molecules in the liver and kidneys, thus affecting the pharmacokinetic profile of any administered drugs [8]. The efficacy of the ABC inhibitors in increasing drug accumulation mentioned above was never in question, but co-administration of these inhibitors has often led to increased toxicity, likely due to the increased drug accumulation that is not adequately cleared [8]. The presence of ABC transporters across most physiological barriers throughout the body impacts the pharmacokinetics of anticancer drugs, especially drugs that rely on renal clearance. Therefore, advanced strategies need to be considered when it comes to ABC inhibition. In other words, the inhibition of ABC transporters has to be specific at the BBB and the GBM tumor mass. For example, aptamers can be used to specifically target resistant GBM tumors. Small and specific, aptamers are single-stranded DNAs or RNAs that bind to the target with high affinities [87]. The specificity of aptamers can be leveraged to target tumor masses that overexpress ABC transporters to potentially deliver ABC inhibitory signals [88]. Nanodelivery could similarly be exploited to improve drug delivery to GBM tumors. Utilizing nanoparticles would allow drugs to bypass the BBB entirely and avoid the dreaded ABC transporters while simultaneously potentially carrying ABC inhibitors or silencing signals for the ABC transporters to improve drug accumulation in the tumor masses.

The fact that ABCB1 was found to export every tested drug (TMZ, etoposide, topotecan, doxorubicin, carmustine, irinotecan, and aldoxorubicin) bar vincristine suggests that studies looking into ABC transporters should make ABCB1 the number one priority in their studies. However, studies that are using TMZ as their chemotherapeutic drug need to be aware of the lack of ligand specificity for multiple ABC transporters, possibly requiring the inhibition of multiple ABC transporters. Even for studies looking into drugs, it would behoove researchers to look into the inhibition of multiple ABC transporters to eliminate any redundant nature of the drug exporters. Besides the ABC transporters that were listed in Table 2 and Table 3, preclinical validations would be required for the ABC transporters that were listed in Figure 5, specifically for ABCA1, ABCA13, and ABCD4. While several studies reported on the mechanisms that regulate the expression of ABC transporters, there are still multiple gaps that would require further attention. The PI3K/Akt/NF-κB pathway and GBM stem cells seemed to be the common strings that tied the expression of most ABC transporters that were studied, but further studies would be required to uncover the mechanisms behind ABC transporter activations. By elucidating the specific mechanisms behind ABC expression, a firmer grasp can be had on the relationship between an individual’s responses to drugs at the genetic level, opening the pathway for the identification of specific pathways or mechanisms that are susceptible to an increased MDR phenotype.

There are limitations to this scoping review that have to be acknowledged. The most obvious limitation is the narrow range of ABC transporters that were actually included in this study. A more detailed recount of the inhibition of a wider range of ABC transporters on drug efficacy would be ideal. Perhaps we were limited by the insistence on including studies that included both in vitro and in vivo studies to explore the translation of said inhibitions of ABC transporters in a GBM environment more akin to one found in patients, but in doing so, more studies will inadvertently be left unexplored on the table. To make this review more feasible, the drug search in the proposed search terms included the most common chemotherapeutic drug used in GBM treatment. As such, this may also have limited the number of potential studies that could have been further included. Additionally, constraints surrounding the period of conceptualization of this paper, only original articles up to 2021 were included. Nevertheless, recent works are all in agreement with the conclusion proposed, with the suppression of ABC transporters such as ABCA1 [71], ABCC1 [89], ABCG2 [90], and ABCB1 [90,91,92,93,94] improving the efficacy of TMZ treatment in GBM. The fact that the suppression of ABCB1 also coincided with increased efficacy of several other drugs such as vincristine [92], etoposide [92], and doxorubicin [95] only strengthens ABCB1 as a therapeutic target. Zhang et al. also corroborated the involvement of moesin in facilitating the assembly of ABC transporters in the form of ABCB1 [94]. With that said, this review still provided valuable insights on the importance of highlighting ABC transporters in the chase for better survival outcomes for patients with GBM.

## 4. Materials and Methods

### 4.1. Literature Search Strategy

The Preferred Reporting Items for Systematic Reviews and Meta-Analyses (PRISMA) statement was used to design, analyze, and report data throughout this review [96]. A literature search was performed across 5 electronic databases (PubMed, ScienceDirect, Ovid Medline, Ovid Embase, and Scopus) where only English articles, published from January 2011 up till June 2021, were included. The following search terms and Boolean connectors were included in the search strategy in the abstract, title, or keywords: ‘Glioblastoma OR malignant glioma’ AND ‘ABC transporter OR p-glycoprotein’ AND ‘TMZ OR procarbazine OR irinotecan OR vincristine OR carmustine OR lomustine’.

### 4.2. Study Selection

The process was facilitated using Covidence [97]. Once the duplicates were removed, the selection of studies was initially performed by screening the abstracts of the published papers that were obtained from the search. Two independent authors reviewed the eligibility of the articles to be included in this study, with an independent third reviewer resolving any discrepancies between the two authors. Once the studies were selected, it was followed by full-text reviews and data extraction, which were performed by two independent authors, based on the inclusion and exclusion criteria of this study. The inclusion criteria included studies that involved (i) human GBM cell lines; (ii) inhibitors of ABC transporters; and (iii) knockdown or downregulation of ABC transporters in in vitro and in vivo models. Studies were excluded if non-human GBM cell lines were used. Studies were also excluded if only in vitro studies were reported to ensure that the in vitro findings we discuss have clear potential for translation to in vivo settings, thus maximizing the likelihood of future research progressing beyond the preclinical phase.

### 4.3. Data Abstraction and Synthesis

The data-charting form was devised as a cooperative effort by the two reviewers. The two reviewers independently read each document and extracted the relevant results. Any discrepancies in the data-extraction process were resolved and discussed with the third independent reviewer, where the form is then subsequently updated iteratively on the data-charting form. Data from eligible studies were charted using a standardized data sheet tailored for this study. Relevant data extracted include in vitro cell lines used, types of drugs used, usage of ABC inhibitors, involved signaling pathways reported by the authors, inhibition or knockdown of ABC transporter genes either in vitro or in vivo, in vitro viability measurements, and in vivo tumor growth parameters. We grouped the studies based on the use of in vitro or in vivo models and summarized the effect on drug efficacy when the ABC transporters in question were inhibited along with more broad findings.

### 4.4. Assessment of Risk of Bias

The quality of the studies included was assessed using SYRCLE’s risk of bias tool for animal studies [98] (Supplementary File S1). Two independent authors evaluated the quality of this study based on the criteria laid out in the risk of bias tool. Any disagreements or conflicts were resolved through the involvement of a third independent reviewer.

### 4.5. Survival Analysis

Level three gene expression profiles of The Cancer Genome Atlas (TCGA) patients with the GBM cohort were obtained from UCSC Xena (https://xenabrowser.net/datapages/) (accessed on 1 June 2021) [99]. Specifically, AgilentG4502 microarray log2-transformed data of 585 patients were obtained from UCSC Xena. Clinical data of the patients, such as overall survival, survival status, IDH1 mutation status, and the type of therapy received, were obtained from cBioPortal (https://www.cbioportal.org/) (accessed on 2 June 2021) [100]. Patients lacking information regarding the overall survival and survival status were excluded from the analysis. Only patients who survived more than 30 days were included to ensure that patients received at least one round of treatment, leaving a final number of 509 patients that were included in the analysis. Patients were dichotomized into high- and low-expression groups based on the median expression. Overall survival of patients between high and low ABC transporter expressions was compared using the log-rank test. This was performed in RStudio v4.1.1 using the ‘survival’ [101] and ‘survminer’ packages. A *p*-value of <0.05 denotes a statistically significant result. A multivariate survival analysis was carried out between the genes that were statistically significant in the univariate Cox regression analysis.

### 4.6. Microarray Differential Expression Analysis

Log2 counts of 7 different microarray datasets containing expression data of patients with GBM and normal brain samples were downloaded from GEO (https://www.ncbi.nlm.nih.gov/geo/) (accessed on 2 June 2021). Differential expression analysis was performed in RStudio v4.1.1 using the limma package. |Log2 fold change (LFC)| > 0.6 and Benjamini–Hochberg adjusted *p*-values (padj) < 0.05 were set as the cut-offs for the screening of differentially expressed genes. Subsequently, ABC transporter genes that meet the cut-off thresholds are then further extracted for further analysis.

## 5. Conclusions

There can be no denying that cancer treatment in general is a challenging prospect, doubly so for patients with GBM. This review has laid out paradigms about the clinical significance of ABC transporters, with multiple ABC transporters at the forefront of increased drug resistance in GBM, with ABCB1 and ABCG2 being the most widely reported among the transporter family. These two transporters are no doubt major drivers of patients with GBM’s drug resistance, but the functionally overlapping nature of ABC transporters needs to be kept in mind. On that note, there exists a knowledge gap, and branching out across the ABC transporter family tree, such as ABCA1, ABCA13, ABCB9 and ABCD4 which carry negative prognostic values in GBM therapy evaluations using alkylating agents, might root out new discoveries. Additionally, the clinical therapeutic and prognostic significance of ABCB5 with reduced hazard risk should be further investigated. Nevertheless, attention should be given to the drug–ligand specificity issue to reduce the burden of acquired drug resistance in GBM. The efficacy of currently existing therapies could be improved upon by a more elegant brute force approach, increasing drug exposures in patients without any dosage increase, and alleviating further dosage-related hematologic side effects. The next monumental breakthrough in oncology could still take decades, but working towards improving the current existing therapies by way of ABC transporters could improve patients with GBM’s quality of life in the near future.

## Figures and Tables

**Table 1:** In vitro and in vivo experimental setups of the 17 included studies.

Papers	Cell Lines	ABC Transporters	Drugs
In Vitro			
Torres et al. [31]	U-87MG, PDC	ABCC1	Vincristine
Ros et al. [32]	U-87MG, A-172, T98G	ABCB1	Temozolomide, Aldoxorubicin
Zhang et al. [33]	U-87MG, A-172	ABCE1	Temozolomide
Fujihara et al. [34]	U-87MG, U-251MG, PDC	ABCB1	Temozolomide
Zhang et al. [35]	U-87MG, U-251MG, PDC	ABCG2	Temozolomide
Li et al. [36]	U-87MG, LN-229	ABCC1	Temozolomide
Jiang et al. [37]	U-87MG, LN443, A-172, U118, PDC	ABCB1	Irinotecan, Pitavastatin
Lee et al. [38]	LN-18, LN-229, U-87MG	ABCB5	Temozolomide
Munoz et al. [39]	U-87MG, T98G	ABCB1	Temozolomide, Erlotinib
Chou et al. [40]	U-87MG, GBM 8401, U-251MG	ABCB1	Carmustine, Doxorubicin
Tso et al. [41]	PDC	ABCB1, ABCG2	Temozolomide
Salaroglio et al. [42]	PDC	ABCB1	Temozolomide, Doxorubicin, Etoposide, Topotecan, Irinotecan
Mujumdar et al. [43]	PDC	ABCB1	Temozolomide, Doxorubicin
Jeon et al. [44]	A-172, A1207, LN-18, LN-229, PDC	ABCA2, ABCC2, ABCC3, ABCC6, ABCG2	Carmustine
**In Vivo**			
Torres et al. [31]	U-87MG	ABCC1	Vincristine
Ros et al. [32]	U-87MG	ABCB1	Temozolomide + Aldoxorubicin
Zhang et al. [33]	U-87MG	ABCE1	Temozolomide
Fujihara et al. [34]	U-87MG	ABCB1	Temozolomide
Zhang et al. [35]	U-251MG	ABCG2	Temozolomide
Li et al. [36]	U-87MG	ABCC1	Temozolomide
Jiang et al. [37]	U-87MG	ABCB1	Pitavastatin, Irinotecan
Lee et al. [38]	U-87MG, LN229	ABCB5	Temozolomide
Munoz et al. [39]	U-87MG, T98G	ABCB1	Temozolomide, Erlotinib
Chou et al. [40]	U-87MG, GBM8401	ABCB1	Carmustine
Tso et al. [41]	PDC	ABCB1, ABCG2	Temozolomide
Salaroglio et al. [42]	PDC	ABCB1	Temozolomide
Mujumdar et al. [43]	PDC	ABCB1	Temozolomide
Jeon et al. [44]	A1207	ABCC3, ABCC6	Carmustine
Raub et al. [45]	U-87MG	ABCB1	Abemaciclib, Palbociclib
Parrish et al. [46]	PDC	ABCB1, ABCG2	Temozolomide, Rucaparib

**Table 2:** In vitro effect of ABC transporter expression and activity on drug efficacies across the included studies.

References	ABC Inhibitors	ABC Expression/Activity	Drug Efficacy
Torres et al. [31]	AOPCP, MRS1220, LY294002, PD980589	~30% to 40% decrease in ABCC1 in adherent and neurosphere U-87MG and patient-derived cells	Induced cell death increased by around 30–55% in the presence of vincristine and the inhibitors
Ros et al. [32]	-	Combination of TMZ and Aldoxorubicin consumed ATP equal to or less than basal control and verapamil-positive control (calcium channel blocker)	Combination of both drugs increased cell death by around 30–50% in all three cell lines
Zhang et al. [33]	ABCE1 shRNA	*ABCE1* downregulated via shRNA	Presence of ABCE1 shRNA decreased TMZ IC_50_ by ~50%
Fujihara et al. [34]	Ad-DKK3	Ad-DKK3 decreased *ABCB1* mRNA expression by about 10-fold	Presence of Ad-DKK3 and TMZ further increased cell death by around 15–25% in the different cell lines
Zhang et al. [35]	Fasudil	*ABCG2* expression in TMZ-resistant cells was downregulated upon treatment with Fasudil Protein levels of ABCG2 on the cytomembrane and cytoplasm were also decreased	Presence of Fasudil greatly decreased the IC_50_ of TMZ by about 90% in various cells
Li et al. [36]	miR-1268a	miR-1268a decreased ABCC1 expression at the protein and mRNA levels	Presence of miR-1268 + TMZ-induced increased cell death compared to TMZ alone
Jiang et al. [37]	Pitavastatin	Combination of irinotecan and pitavastatin attenuated production of function ABCB1. GBM cells showed increased intracellular amounts of ABCB1 substrate upon pitavastatin treatment	Irinotecan IC_50_ was decreased by 58% in the presence of pitavastatin
Lee et al. [38]	ABCB5 mAB	*ABCB5* expression was inhibited using ABCB5 mABs	20% more cells were detected to be in early + late apoptotic stage when treated with TMZ + ABCB5 mAB compared to TMZ alone
Munoz et al. [39]	ABCB1 siRNA	TMZ reduced subcellular active ABCB1 while showing concomitant increase of active ABCB1 in the cell membrane by activating EGFRs	Inhibition of ABCB1 through direct siRNA knockdown induced increased cell death by around 15~20% compared to TMZ alone EGFR inhibitor erlotinib further increased cell death by 15–20% when treated concomitantly with TMZ
Chou et al. [40]	Tariquidar	Cycling hypoxia increased *ABCB1* mRNA expression by about 5 folds. ABCB1 substrate efflux was increased in the presence of cycling hypoxia	Cells experiencing cycling hypoxia had lower cell deaths when treated with doxorubicin and carmustine. The presence of Tariquidar that inhibits ABCB1 managed to reverse cycling hypoxia-induced survival in GBM cells
Tso et al. [41]	BMP7	Treatment with BMP7 decreased *ABCB1* and *ABCG2* mRNA expression by more than 90%	Presence of BMP7 and TMZ increased cell death by 40%
Salaroglio et al. [42]	CAXII Inhibitor	Presence of inhibitor decreased ABCB1 activity by 80% while TMZ alone decreased ABCB1 activity by 45% Presence of CAXII inhibitor showed increased TMZ accumulation that is comparable to ABCB1 knockout	The presence of CAXII inhibitor and TMZ, doxorubicin, epotoside, and topotecan induced 40–70% more cell death in neurospheres.
Mujumdar et al. [43]	CAXII Inhibitor	CAXII inhibitor decreased ABCB1 ATPase activity by around 60% and increased intracellular doxorubicin and TMZ by 3.2 and 1.32 folds, respectively, in neurospheres	Presence of CAXII inhibitor further increased cell death by around 40–45% compared to TMZ and doxorubicin alone
Jeon et al. [44]	sh-ID4	ID4 overexpression induced the expression of ABCC3, ABCC6 and ABCA2 in A-172 cells shID4 decreased mRNA expression of *ABCC2, ABCC3, ABCC6, and ABCA2* in LN229 cells *shID4 decreased mRNA expression of ABCC3, ABCC6 and ABCG2 in patient-derived GBM cells* *SOX2* overexpression induced the expression of *ABCC3* and *ABCC6*	Overexpression of SOX2 decreased the amount of cell death induced by carmustine shID4 + carmustine further increased cell death by about 20% compared to carmustine alone

**Table 3:** In vivo effect of ABC transporters on drug efficacies across the included studies.

References	ABC Inhibitors	ABC Expression/Activity	Drug Efficacy
Torres et al. [31]	MRS1220	ABCC1 immunohistochemical staining decreased by 0.25-fold upon treatment with MRS1220	Tumor size 17 days post inoculation in the presence of vincristine and MRS1220 further decreased by around 30% compared to vincristine alone
Ros et al. [32]	-	-	Tumor sizes treated with both drugs were 9-fold smaller compared to the controls, while tumor size was 4-fold and 11-fold smaller when treated with TMZ and aldoxorubicin alone, respectively. Both drugs improved median survival by 37.5% and delayed mortality. Aldoxorubicin alone improved survival by 12.5% while TMZ improved survival by 37.5%.
Zhang et al. [33]	ABCE1 shRNA	-	ABCE1 shRNA decreased tumor volume and weight by 27 and 50%, respectively
Fujihara et al. [34]	Ad-DKK3	Immunohistochemical staining showed lower expressions of ABCB1	Combination of Ad-DKK3 and TMZ further decreased tumor weight at day 21 by 61% when compared to TMZ alone Furthermore, 100% of mice treated with Ad-DKK3 + TMZ survived past 28 days while only 65% of mice treated with TMZ survived.
Zhang et al. [35]	Fasudil	Immunohistochemical staining showed treatment with Fasudil correlated with decreased ABCG2 on the membrane	Presence of fasudil and TMZ further decreased tumor volume and weight by about 60 and 49%, respectively. Median survival of mice treated with TMZ + fasudil were further increased by 56% compared to TMZ alone
Li et al. [36]	miR-1268a		TMZ + miR-1268a further decreased tumor weight by 60% compared to TMZ alone
Jiang et al. [37]	Pitavastatin	-	Tumor volume was further decreased by 75% when both drugs were administered compared to their singly administered counterpart
Lee et al. [38]	ABCB5 mAB	-	Tumor volume was further decreased by 29% when treated with ABCB5 mAB + TMZ compared with TMZ alone
Munoz et al. [39]	Erlotinib	-	Concomitant treatment of erlotinib + TMZ further decreased tumor volume by 68% compared with TMZ alone
Chou et al. [40]	YC-1	*ABCB1* transcriptional activation was reduced by half after treatment with XR9576	Mice that received YC-1 that blocks HIF-1α prior to carmustine treatment had better survival rates.
Tso et al. [41]	BMP7	-	Presence of BMP7 and TMZ increased survival by around 24~31% compared to TMZ alone
Salaroglio et al. [42]	CAXII Inhibitor	CAXII inhibitor reduced ABCB1 protein expressions	Presence of CAXII inhibitor and TMZ further decreased tumor volume by around 87% when compared to TMZ alone. Presence of CAXII and TMZ also improved median survival by around 14–50% compared to TMZ alone in different PDC xenografted mice
Mujumdar et al. [43]	CAXII Inhibitor	-	TMZ and CAXII inhibitor further decreased tumor volume at day 30 by around 22~100% compared to TMZ alone in different PDC xenografted mice TMZ and CAXII inhibitors also further increased median survival by 12–40% in different PDC xenografted mice
Jeon et al. [44]		*ABCC3* and *ABCC6* mRNA levels correlated with *ID4* levels	Overexpression of *SOX2* and *ID4* increased tumor weight by around 80% compared to carmustine alone
Raub et al. [45]		-	Unbound partition coefficient was 2.8- and 21-fold higher for palbociclib and abemaciclib, respectively, in *ABCB1* deficient mice
Parrish et al. [46]	-	-	*ABCB1* and *ABCG2* knockout increased rucaparib brain-to-plasma ratio by about 15-fold

**Table 4:** Combined in vitro and in vivo data of the included studies.

ABC Transporters	ABC Inhibitors	Experimental Setup	ABC Expression/Activity	Drug Efficacy	References
Temozolomide					
ABCB1	BMP-7	In Vitro	↓	↑	Tso et al. [41]
		In Vivo	NA	↑	
	CAXII Inhibitor	In Vitro	↓	↑	Salaroglio et al. [42]
		In Vivo	↓	↑	
	CAXII Inhibitor	In Vitro	↓	↑	Mujumdar et al. [43]
		In Vivo	NA	↑	
	Ad-DKK3	In Vitro	↓	↑	Fujihara et al. [34]
		In Vivo	↓	↑	
	ABCB1 siRNA	In Vitro	↓	↑	Munoz et al. [39]
	Erlotinib	In Vivo	↓	↑	
ABCG2	BMP-7	In Vitro	↓	↑	Tso et al. [41]
		In Vivo	NA	↑	
	Fasudil	In Vitro	↓	↑	Zhang et al. [35]
		In Vivo	↓	↑	
		In Vivo	NA	↑	
ABCE1	ABCE1 shRNA	In Vitro	↓	↑	Zhang et al. [33]
		In Vivo	NA	↑	
ABCC1	miR-1268a	In Vitro	↓	↑	Li et al. [36]
		In Vivo	NA	↑	
		In Vivo	NA	↑	
ABCB5	ABCB5 mAB	In Vitro	↓	↑	Lee et al. [38]
		In Vivo	↓	↑	
		In Vivo	NA	↑	
Vincristine					
ABCC1	MRS1220	In Vitro	↓	↑	Torres et al. [31]
		In Vivo	↓	↑	
Carmustine					
ABCC3, ABCC6	Sh-ID4	In Vitro	↓	↑	Jeon et al. [44]
	-	In Vivo	↑	↓	
ABCB1	Tariquidar	In Vitro	NA	↑	Chou et al. [40]
	YC-1	In Vivo	↓	↑	
Irinotecan					
ABCB1	Pitavastatin	In Vitro	↓	↑	Jiang et al. [37]
		In Vivo	NA	↑	
Temozolomide + Aldoxorubicin					
ABCB1	-	In Vitro	↓	↑	Ros et al. [32]
	-	In Vivo	NA	↑	

**Table 5:** Number of GBM samples and healthy brain samples in each GEO microarray dataset.

GEO Dataset	GBM Samples	Healthy Brain Samples
GSE4290 [47]	81	23
GSE15824 [48]	15	2
GSE16011 [49]	159	8
GSE49412 [50]	5	1
GSE50161 [51]	34	13
GSE90604 [52]	16	6
GSE108474 [53]	33	17

**Table 6:** Differentially expressed ABC transporter genes listed in Figure 4 when comparing GBM samples to healthy brain samples.

GEO Datasets	Upregulated	Downregulated
GSE4290 [47]	*ABCA1*, *ABCA13*	*ABCB9*
GSE15824 [48]	*ABCA1*, *ABCD4*	-
GSE16011 [49]	*ABCA1*, *ABCD4*	*ABCB9*
GSE49412 [50]	*ABCA1*	-
GSE50161 [51]	*ABCA1*	*ABCB9*
GSE90604 [52]	*ABCA1*, *ABCD4*	-
GSE108474 [53]	*ABCA13*, *ABCB9*, *ABCB5*, *ABCD4*, *ABCG5*	

## Data Availability

All data used in this study are publicly available on https://xenabrowser.net/datapages/ (accessed on 1 June 2021) and https://www.ncbi.nlm.nih.gov/geo/ (accessed on 2 June 2021).

## Supplementary Materials

The following supporting information can be downloaded at: https://www.mdpi.com/article/10.3390/ph18010102/s1, Table S1: Differential expression analysis for ABC Transporter genes in GSE4290; Table S2: Differential expression analysis for ABC Transporter genes in GSE15824; Table S3: Differential expression analysis for ABC Transporter genes in GSE16011; Table S4: Differential expression analysis for ABC Transporter genes in GSE49412; Table S5: Differential expression analysis for ABC Transporter genes in GSE50161; Table S6: Differential expression analysis for ABC Transporter genes in GSE90604; Table S7: Differential expression analysis for ABC Transporter genes in GSE108474. File S1: Risk of Bias.

## Abbreviations

The following abbreviations are used in this manuscript:

MDPI	Multidisciplinary Digital Publishing Institute
GBM	Glioblastoma
TMZ	Temozolomide
ATP	Adenosine Triphosphate
ADP	Adenosine Diphosphate
MDR	Multidrug Resistance
ABC	ATP-Binding Cassette
PI3K	Phosphoinositide 3-Kinase
Akt	Ak Strain Transforming
NF-κB	Nuclear Factor Kappa B
BBB	Blood–Brain Barrier
BRCP	Breast Cancer Resistance Protein
MGMT	O6-methylguanine-DNA methyltransferase
miRNA	MicroRNA
DNA	Deoxyribonucleic Acid
GST	Glutathione S-Transferases
CYP450	Cytochrome P450
PRISMA	Preferred Reporting Item for Systematic Reviews and Meta-Analyses
PDC	Patient-Derived Cultures
IC50	Half-Maximal Inhibitory Concentration
O_2_	Oxygen
CO_2_	Carbon Dioxide
AOPCP	α,β-Methyleneadenosine 5′-diphosphate
shRNA	Small Hairpin RNA
DKK3	Dickkopf 3
SFRP4	Secreted Frizzled-Related Protein 4
mAB	Monoclonal Antibody
XR9576	Tariquidar
BMP7	Bone Morphogenetic Protein 7
CAXII	Carbonic Anhydrase XII
ID4	Inhibitor of DNA Binding 4
EGFR	Epidermal Growth Factor Receptor
SOX2	SRY-Box Transcription Factor 2
TGF-β	Transforming Growth Factor Beta
HIF-1α	Hypoxia-Inducible Factor 1-Alpha
JNK	Jun N-terminal Kinase
ERK	Extracellular Signal-regulated Kinase
EGF	Epidermal Growth Factor
c-Jun	Jun Proto-Oncogene
c-FOS	FOS Proto-Oncogene
ROCK2	Rho-Associated Kinase 2
SMAD	Suppressor of Mothers against Decapentaplegic
TCGA	The Cancer Genome Atlas
HR	Hazard Ratio
GEO	Gene Expression Omnibus
CD133	Prominin-1
LFC	Log_2_ Fold Change
