# Supplementary material for: Revisiting ABC Transporters and Their Clinical Significance in Glioblastoma
Source: Pharmaceuticals (Basel). 2025 Jan 15;18(1):102. doi: 10.3390/ph18010102 (PMC11769420; doi:10.3390/ph18010102)
Supplement: Supplementary file 1 [file pharmaceuticals-18-00102-s001.zip › pharmaceuticals-3388539-supplementary/File S1Risk of Bias.pdf]

**Studies**	**1**	**2**	**3**	**4**	**5**	**6**	**7**	**8**	**9**	**10**	**Overall**
Torres *et al.* 2016 [30]	Y	Y	Y	Y	Y	N	UC	Y	UC	N	Fair
Ros *et al.* 2018 [31]	Y	Y	Y	Y	Y	Y	UC	Y	Y	N	Good
Zhang *et al.* 2018 [32]	Y	Y	Y	N	UC	N	UC	Y	Y	Y	Fair
Fujihara *et al.* 2018 [33]	Y	Y	Y	Y	Y	Y	UC	Y	Y	N	Good
Zhang *et al.* 2018 [34]	Y	Y	Y	Y	Y	Y	UC	Y	Y	N	Good
Li *et al.* 2018 [35]	U	Y	U	U	N	U	Y	Y	Y	Y	Fair
Jiang *et al.* 2014 [36]	Y	Y	Y	N	UC	N	UC	Y	Y	Y	Fair
Bhuvanalashmi *et al.* 2015 [37]	Y	Y	Y	UC	UC	N	UC	Y	Y	Y	Fair
Lee *et al.* 2020 [38]	Y	Y	Y	Y	Y	Y	UC	Y	Y	N	Good
Munoz *et al.* 2014 [39]	Y	Y	Y	N	UC	N	UC	Y	Y	Y	Fair
Chou *et al.* 2012 [40]	Y	Y	Y	Y	Y	Y	UC	Y	Y	N	Good
Tso *et al.* 2015 [41]	Y	Y	Y	N	UC	N	UC	Y	Y	Y	Fair
Salaroglio *et al.* 2021 [42]	Y	Y	Y	Y	Y	Y	UC	Y	Y	N	Good
Mujumdar *et al.* 2019 [43]	Y	Y	Y	Y	Y	Y	UC	Y	Y	N	Good
Jeon *et al.* 2011 [44]	Y	Y	N	N	UC	N	UC	N	Y	Y	Fair
Raub *et al.* 2015 [45]	Y	Y	Y	Y	Y	Y	UC	Y	Y	N	Good
Parrish *et al.* 2015 [46]	Y	Y	Y	Y	Y	Y	UC	Y	Y	N	Good

Questions

1. Was the allocation sequence adequately generated and applied?

2. Were the groups similar at baseline or were they adjusted for confounders in the analysis?

3. Was the allocation adequately concealed?

4. Were the animals randomly housed during the experiment?

5. Were the caregivers and/or investigators blinded from knowledge which intervention each animal received during the experiment?

6. Were animals selected at random for outcome assessment?

7. Was the outcome assessor blinded?

8. Were incomplete outcome data adequately addressed?

9. Are reports of the study free of selective outcome reporting?

10. Was the study apparently free of other problems that could result in high risk of bias?
